# Impact of multiple consultations and switching in treatment-seeking in tuberculosis: Mathematical modelling and optimal control

**DOI:** 10.1371/journal.pone.0324330

**Published:** 2025-06-10

**Authors:** Preeti Saini, Samit Bhattacharyya

**Affiliations:** Disease Modelling Lab, Department of Mathematics, School of Natural Sciences, Shiv Nadar Institution of Eminence (SNIoE), NH-91, Greater Noida, Uttar Pradesh, India; Texas A&M University College Station, UNITED STATES OF AMERICA

## Abstract

Treatment-seeking behaviour significantly contributes to the worldwide tuberculosis (TB) burden, especially in Southeast Asia and African regions. At the onset of symptoms like coughing, fever, fatigue, and loss of appetite, individuals visit various clinics or alternative medicine centres, often switching multiple times before actually reaching a TB DOTS (Directly Observed Therapy Short Courses) centre. This, however, introduces a long delay in proper diagnosis and treatment of TB cases, which increases community transmission and the overall TB burden. By synthesizing data from various empirical studies, we develop an intricate mathematical model of such multiple consultations and aim to quantify the impact of such behavioural interactions on disease burden. Our SIR-based TB transmission framework quantifies the rise in active TB cases due to delays from multiple consultations before diagnosis and treatment at DOTS centres. We found that up to 2-3 consultations before diagnosis can substantially lower the overall TB burden. Using optimal control modelling, we propose targeted interventions – including enhanced TB awareness, early detection, and improved healthcare infrastructure. These findings offer valuable insights for policymakers and public health organizations to develop effective strategies for TB control in high-prevalent regions.

## Introduction

**Fig 1:**
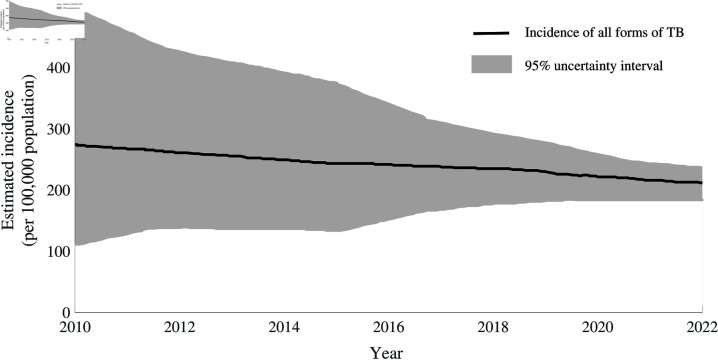
Estimated incidence of all forms of TB in India per 100,000 population as per India TB report 2024 [5].

Within the realm of infectious diseases, tuberculosis, despite its historical roots and the accessibility of affordable treatments, presently stands as the predominant cause of death. Annually, the global incidence of TB exceeds 10 million cases, with a mortality toll surpassing 1 million [1, 2]. The South-East Asian Region, Africa, and the Western Pacific had 46%, 23%, and 18% respectively, of newly reported TB cases in 2021. The top 30 nations with the highest TB burden account for 87% of all TB cases [3]. India contributes significantly to the global TB burden, accounting for an estimated 28% of global TB cases and 26% of global TB deaths in 2023 [4]. In 2023, India reported 2.55 million TB cases, marking the highest number ever, although the incidence rate declined by 17.7% from 237 in 2015 to 195 per 100,000 population in 2023 (Fig 1). This substantial burden not only has an impact on individual health but also poses a formidable obstacle to national as well as global endeavours in TB control.

The transmission of TB in India is influenced by numerous factors, including a high prevalence of malnutrition, socioeconomic disparities, and the interplay between weakened immune systems and HIV/AIDS [6, 7]. Beyond such factors, individual treatment-seeking behaviour also significantly hampers the effectiveness of global TB control programs [8]. Patients experiencing symptoms often initially seek assistance from general practitioners or pharmacists at local medical stores. However, if symptoms persist, they typically transition to seeking care from qualified healthcare professionals, such as clinics and certified medical practitioners [9–11]. This behaviour not only affects individual disease outcomes but also impacts community transmission and mortality rates [12–15].

**Fig 2:**
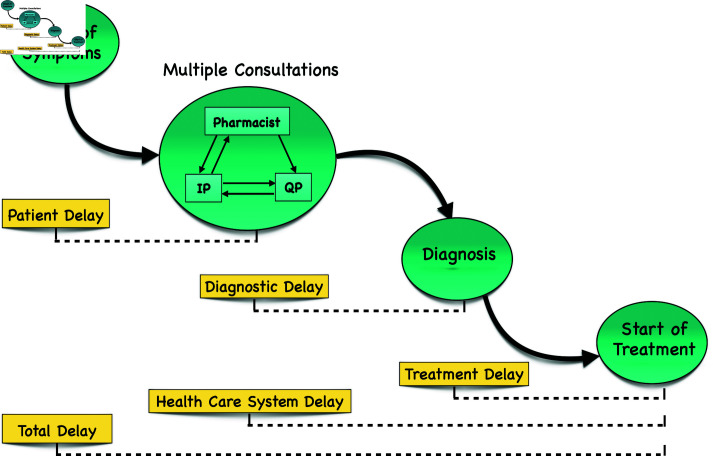
Types of delay in treatment-seeking pathway of TB. IP and QP stand for Informal Provider and Quality Provider. See text for details.

In general, three types of delay may occur in the entire treatment-seeking pathway – from the onset of developing symptoms to proper treatment [16, 17] (Fig 2): (i) *patient delay*, which pertains to the time interval between the emergence of symptoms and the initial interaction with a healthcare professional; (ii) *diagnostic delay*, encompassing the period from the first healthcare encounter to the accurate diagnosis of TB; and (iii) *treatment delay* that denotes the duration between the TB diagnosis and the commencement of TB treatment [18, 19].

Numerous survey studies have been conducted to assess the extent to which such delays occur. A study conducted in the Patna district of India, based on interviews with 64 self-reporting TB patients, found that the mean total delay in receiving TB care was 40 days, with diagnostic delay accounting for 58% of this duration [20]. Similarly, a systematic review by Bello *et al*. (2019) on TB treatment-seeking behaviour across 78 countries observed that patient delay was the largest contributor, averaging 81 days out of a mean total delay of 87.6 days. Diagnostic delay accounted for a mean of 29.5 days, while treatment delay contributed an additional 7.9 days [21]. Another study conducted among pulmonary TB patients at the Government Chest Clinic in Nigeria reported a median patient delay of 60 days [22]. While many studies [23–25] highlight that socio-demographic, socioeconomic, and sociocultural factors significantly influence the type and duration of delays, other research [9, 12, 26, 27] underscores a critical issue: the cyclical pattern of repeated visits to the same level of healthcare, which perpetuates delays in TB treatment-seeking behaviour.

Mathematical models have significantly enhanced our understanding of dynamics of the TB incidence in population, TB epidemiology and cost-effective intervention in population. For example, studies include case detections and genetic susceptibility [28], estimating TB incidence in India [29], impact of reinfections [30], vaccine efficacy [31], optimal intervention including specific testing and treatment strategies [32–34], effect of population demographic and comorbidity [35], co-circulation of drug-sensitive and drug-resistant TB [36] and such others. While numerous recent studies have examined care-seeking behaviour for diseases such as COVID-19 [37] and epilepsy [38], relatively few have focused on modelling patient behaviour and its impact on the TB burden within communities. One notable example is the study by Deo *et al*. [39], which developed a simulation-based model of patients’ diagnostic pathways. This model captures key behavioural characteristics of both healthcare providers (e.g., time to order a diagnostic test) and patients (e.g., time to switch to another provider) and examines their effects on TB transmission dynamics. Using quantitative data from Mumbai and Patna, the study concluded that fostering public-private partnerships in diagnosis and treatment during patient consultations could significantly reduce delays in urban Indian settings. However, no existing studies specifically address delays caused by multiple consultations and patient-switching behaviours or their impact on the overall TB disease burden in the population.

In this paper, we develop a TB transmission framework using the multiple consultations and switching data obtained in the survey studies from Kapoor *et al* (2012) [9]. We incorporate patient behaviour and analyse the model through stochastic implementations and predictions. We also modify the basic model with interventions such as focused campaigns to increase awareness of TB symptoms, encourage early identification, and strategic measures and determine optimal cost-effective control to reduce the impact of delay on the TB burden in population. This analysis offers policy insights for evaluating the efficiency of different control strategies, guiding public health sectors in formulating policy decisions in regions with higher TB prevalence.

## Mathematical model

**Fig 3:**
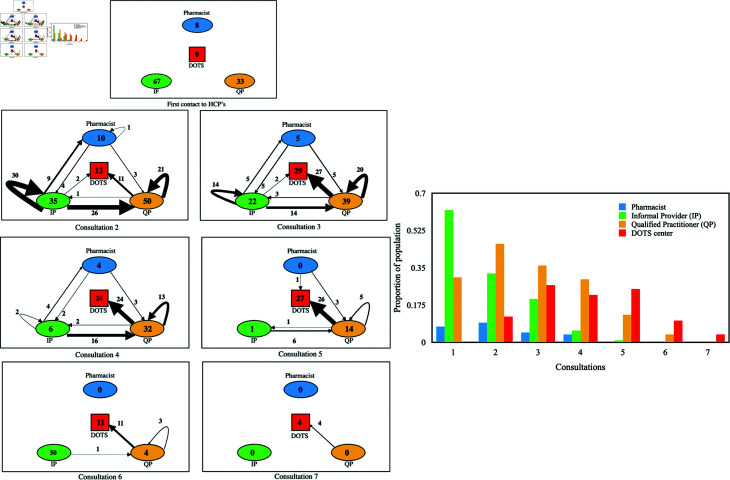
Left panel: Treatment-seeking and switching patterns among 108 patients under a longitudinal survey, showing consultations across Pharmacists, Informal Providers (IP), Qualified Practitioners (QP), and DOTS centres. Seven larger boxes depict switching patterns after seven consultations. Numbers inside circles and squares indicate patients switching to different consultations, while arrow widths represent the magnitude of these transitions. Right panel: Bar graph illustrating the proportion of patients consulting various HCPs (Healthcare Practitioners). (data adapted from [9])

We develop the TB transmission model in the population incorporating the behavioural aspects of multiple consultations and switching in treatment-seeking. In general, it has been observed that individuals seek at least five to seven consultations before actually going to DOTS centre for diagnosis and treatment [9, 40]. Fig 3 depicts one such behavioural instance in a population in Delhi, India [9]. When symptoms first appeared, out of a total of 108 individuals, sought initial consultation from pharmacies, while a minority approached qualified healthcare providers, and the remainder sought assistance from informal providers. Then they switched to different providers upon second and subsequent consultations, and finally, all went to the DOTS centre (Fig 3).

**Fig 4:**
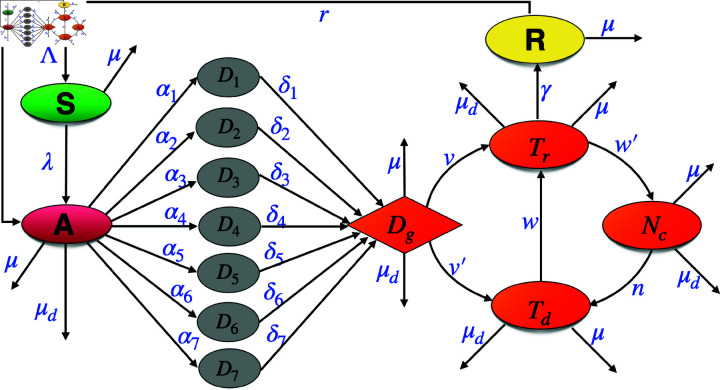
Schematic representation of population dynamics in the model. The total population is *N*(*t*). Susceptible population (*S*(*t*)), upon acquiring infection, moves to the active TB compartment (*A*(*t*)). Active TB patients are experiencing delays through multiple consultations (*D*_*i*_(*t*)), and finally diagnosed in the compartment *D*_*g*_(*t*). Diagnosed patients eventually moves to treatment compartment (*T*_*r*_(*t*)), but those who are delaying the start of treatment, moving to the compartment *T*_*d*_(*t*). The diagnosed TB patients who discontinue their treatment moves to the compartment (*N*_*c*_(*t*)) and those who have successfully recovered from TB disease finally moved to the recovered compartment (*R*(*t*)).

We represent this multiple consultations in treatment-seeking behaviour by categorizing individuals based on the number of consultations they undergo. Specifically, individuals in *D*_*i*_ have *i* consultations before receiving an accurate diagnosis. Our model accounts for up to seven consultations. Here, the total population at time *t*, *N*(*t*) is subdivided into 14 mutually disjoint compartments consisting of susceptible population (*S*(*t*)), active TB patients (who is yet to seek his/her first consultation) (*A*(*t*)), active TB patients before diagnosis in *i* consultation(s) (*D*_*i*_(*t*)), active TB patients diagnosed (*D*_*g*_(*t*)), active TB patients diagnosed and are delaying the start of their treatment (*T*_*d*_(*t*)), diagnosed patients who immediately start their treatment (*T*_*r*_(*t*)), patients who discontinue their treatment at a premature level (*N*_*c*_(*t*)), those who have successfully recovered from TB after the treatment (*R*(*t*)). We assume that $\alpha_{i}$ is the rate of ${\text{i}}^{\text{th}}-$ consultations and $1/\delta_{i}$ is the average period they spent in *D*_*i*_ compartments before actually going to the DOTS center for diagnosis. Detailed descriptions of model variables and parameters are given in Table 1 and 2 respectively.

**Table 1:** Description of variables used in the model

Variable	Description
*S*(*t*)	Susceptible
*A*(*t*)	Active TB patients (who is yet to seek his/her first consultation)
*D*_*g*_(*t*)	Active TB patients diagnosed
*T*_*r*_(*t*)	Diagnosed TB patients with immediate start of treatment
*T*_*d*_(*t*)	Active TB patients diagnosed but with treatment delay
*N*_*c*_(*t*)	Non-compliant TB patients
*R*(*t*)	Recovered TB patients
*D*_*i*_(*t*)	Active TB patients before diagnosis in *i* consultation

**Table 2:** Baseline parameter values for simulation

Parameters	Description	values	ref
Λ	Recruitment rate per day	10	[31]
β	Average number of infections per TB case per day	3/365−11/365	[41]
μ	Natural mortality rate per day	1/(70×365)	[29]
$\mu_{d}$	Disease-related mortality rate per day	0.24/365	[41]
$\alpha_{i}$	Treatment-seeking rate in *i* consultation per day	0–0.7	[42–44]
$\frac{1}{\delta_{i}}$	Duration until proper diagnosis at DOTS center	15–95 days	[18, 45, 46]
*v*	Immediate treatment initiation rate for diagnosed patients per day	0.85	[47]
$v^{\prime }$	Delay rate for diagnosed patients in initiating treatment per day	0.15	Calibrated
*w*	Delayed-treatment initiation rate post-diagnosis per day	0.1	[48]
$w^{\prime }$	Non-compliance rate for patients undergoing treatment per day	0.11	[49]
*n*	Re-initiation rate of treatment for non-compliance per day	0.05	[50]
γ	Rate of recovery per day	0.8	[51]
*r*	Rate of relapse in the recovered individuals per day	0.0033/365	[41]
$\eta_{i}$	Reduced probability of transmission of infection for diagnosed or treated individuals	0.2–0.5	Calibrated

Based on the above assumptions, the model is given by the following system of non-linear ordinary differential equations


$$\frac{dS}{dt}\Lambda -\lambda S-\mu S$$

$$\frac{dA}{dt}=\lambda S-\sum_{1}^{7}\alpha_{i}A+rR-(\mu +\mu_{d})A$$

$$\frac{dD_{i}}{dt}=\alpha_{i}A-\delta_{i}D_{i}\;;\;i=1\text{ to }7$$

$$\frac{dD_{g}}{dt}=\sum_{1}^{7}\delta_{i}D_{i}-(v+v\prime )D_{g}-(\mu +\mu_{d})D_{g}$$

$$\frac{dT_{r}}{dt}=vD_{g}+wT_{d}-(w\prime +\gamma )T_{r}-(\mu +\mu_{d})T_{r}$$

$$\frac{dT_{d}}{dt}=v\prime D_{g}+nN_{c}-wT_{d}-(\mu +\mu_{d})T_{d}$$

$$\frac{dN_{c}}{dt}=w\prime T_{r}-nN_{c}-(\mu +\mu_{d})N_{c}$$

$$\frac{dR}{dt}=\gamma T_{r}-(r+\mu )R$$ (1)

where the force of infection (λ) is given by


$$\lambda =\frac{\beta (A+\sum_{1}^{7}D_{i}+\eta_{1}D_{g}+\eta_{2}T_{d}+\eta_{3}T_{r}+\eta_{4}N_{c})}{N}$$

Here, $\eta_{i}$ represents the reduced probability of transmission of infection for diagnosed, diagnosed but delaying treatment, treated, and non-compliant individuals.

## Stochastic simulations

We employ a stochastic implementation of the underlined deterministic system and simulate the model to have a deeper understanding of the quantitative aspects of the impact of delay. We assume the total population is approximately 100,000 with a few active TB patients. Baseline parameter values are depicted in Table 2. The initial condition is $\left(S=99500,A=500,D_{i}=0,D_{g}=0,T_{r}=0,T_{d}=0,N_{c}=0,R=0\right)$. We simulate the model for 10 years. The details of stochastic implementation are as follows:

We implement using a tau-leap algorithm, which is an event-driven approximated method of the Gillespie algorithm used for the simulation of stochastic systems [65]. A small population, where event-driven stochasticity is more significant, is utilized to quantify the effect of delay on TB disease burden. The unit for the time step of the simulation is day. The algorithm performs a reaction after updating the propensity functions at each interval length τ=day. In this context, it is presumed that all disease and demographic processes (including transmission, diagnosis, treatment, recovery, and reinfection) in the model equations are event-driven.

The summary of the tau leap algorithm is as follows: let *g*(*t*) be a disease compartment. Let *E*_*k*_ be the list of events that occurs at the rate *r*_*k*_(*g*(*t*))(most of our reactions are density dependent) and with a state change matrix *M*_*j*_. First, we initialize the model with the initial condition *g*(*t*_0_) and then calculate the event rates $r_{k}g(t_{0})$. We choose a fixed time step, i.e. τ=1. For each event *E*_*k*_, we then sample $\zeta_{k}$ from a Poisson distribution with mean $\tau r_{k}$. This $\zeta_{k}$ is the number of times each event occurs during the time interval [t,t+τ]. Finally, we update the state by $g(t+\tau )=g(t)+\sum_{i}\zeta_{k}M_{j}$.

## Results

**Fig 5:**
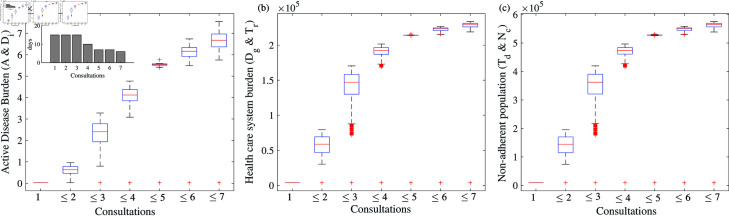
Cumulative incidences of TB disease with multiple consultations: ( **a**) Active disease burden (*A* + *D*_*i*_), ( **b**) health care system burden ($D_{g}+T_{r}$), ( **c**) non-adherent population ($T_{d}\;+\;N_{c}$). We have chosen β=11/365, $\eta_{1}=0.2$, $\eta_{2}=0.4$, $\eta_{3}=0.2,\eta_{4}=0.4$. Values of the other parameters are taken from the Table 2. The figure in the inset indicates the number of days between subsequent consultations (i.e., $1/\delta_{i}\;-\;1/\delta_{i-1}$ = days between *i*th and (*i*–1)*th* consultation). In each case of involving multiple consultations (≥2), box plot is drawn using 1500 samples of $\left(\alpha_{1},\alpha_{2},\ldots ,\alpha_{7}\right)$ with $\sum_{i=1}^{7}\alpha_{i}=0.7$. We have considered 10 years simulation of the model in each scenario. See text for details.

Studies show that the treatment-seeking rate for individuals with active TB across different populations and countries varies from 55–70% (WHO Global TB Report 2024), [4]. In our model simulation, we have assumed treatment-seeking rate is approximately 70% . In each scenario involving multiple consultations (≥2), we generated 1,500 samples of $\left(\alpha_{1},\alpha_{2},\ldots ,\alpha_{7}\right)$ such that the total sum equals 0.7 $(i.e.,\alpha_{i}\geq 0,\sum_{i=1}^{7}\alpha_{i}=0.7$). For example, in Fig 5, for the scenario where the number of consultations is at most three (≤3), we used 1,500 samples of $(\alpha_{1},\alpha_{2},\alpha_{3},0,0,0,0$) ensuring that $\alpha_{1}+\alpha_{2}+\alpha_{3}=0.7$. Fig 5 illustrates a comparative outcome of the disease burden in different compartments when the average number of infections per TB case per day (β) is 11/365 and $\eta_{1}=0.2$, $\eta_{2}=0.4$, $\eta_{3}=0.2,\eta_{4}=0.4$. Other parameters value have been described in Table 2. The time between subsequent consultations has been considered as 15-15-15-10-7-7-6 (inset figure in Fig 5(a)). Altogether the patient delay extend for nearly 75 days (2.5 months) starting from the onset of symptoms. When all the TB patients are immediately diagnosed after the symptoms appear, the burden of active patients (new cases) together in the *A* and *D*_*i*_ compartments is $2.2255\times 10^{4}$ (Fig 5(a)), $3.669\times 10^{3}$ patients in the *D*_*g*_ and *T*_*r*_ compartments (Fig 5(b)) and $9.067\times 10^{3}$ in the *T*_*d*_ and *N*_*c*_ compartments together (Fig 5(c)). However, the disease burden escalates to $7.5455\times 10^{6}$ in active disease population (Fig 5(a)), which is approximately 340-fold increase from the baseline estimation when at most seven consultations are made before properly diagnose. The effect is similar on the other compartments - approximately 64-fold increase in the burden (Fig 5(b) & (c)). This underscores that delaying proper diagnosis and treatment through multiple consultations not only heightens burdens on individuals seeking care but also amplifies the burden on the healthcare system equally.

**Fig 6:**
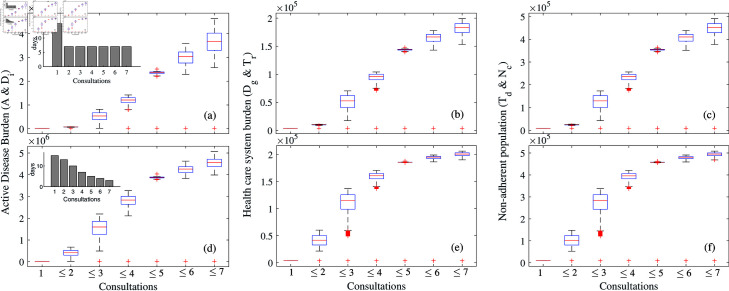
Cumulative incidences of TB disease when the number of days between subsequent consultations is ( a– c) uniform (15-7-7-7-7-7-7) and ( d– f) exponential (15-13-10-7-5-4-3). Left vertical panel (a and d) describes burden on active disease, middle panel (b and d) Healthcare system burden, and (e and f) is burden of non-adherent individuals. See text for details.

We also explored different distributions for the intervals between subsequent consultations, as various survey studies have highlighted their variability [52, 53]. In our simulations, we considered a uniform distribution (i.e., 15-7-7-7-7-7-7 days) and an exponential distribution (i.e., 15-13-10-7-5-4-3 days), with a total of 57 days as patient delay in both cases due to multiple consultations. Fig 6 exhibits the difference in the pattern of disease burden under these different distributions. Although individuals initiate the first consultation around 15 days from the onset of symptoms in both cases, the burden accumulates higher in case of exponential distributions. For example, there is an almost negligible increase in uniform distribution when individuals are properly diagnosed by the end of 2 consultations. This is not the scenario when we consider exponential distribution. Also, if all the active TB patients are properly diagnosed by at most three consultations then the average burden of active patients is $5.39795\;\times \;10^{5}$ in the scenario of the uniform distribution (Fig 6a). In contrast, if we consider as exponential distribution of number of days between subsequent consultation, the burden increases up to $1.599200\;\times \;10^{6}$, which is almost a 3-fold increase from the earlier (Fig 6e). A similar pattern is observed after 4 and subsequent consultations. There is also incremental burden in the healthcare system and non-adherent populations. So, this simulation exhibits that a uniform time gap between consultations is preferable to an exponential one, even though the total patient delay of proper diagnosis is the same (57 days) in both cases.

**Fig 7:**
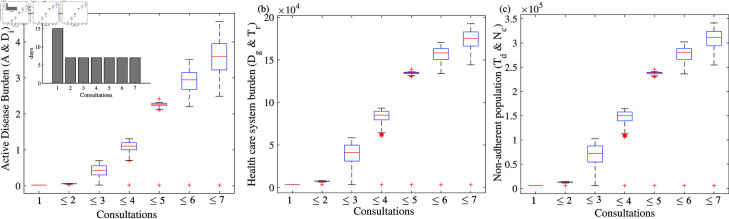
Burden of TB disease when there is only patient delay and no treatment delay and the number of days between subsequent consultations is uniform (15-7-7-7-7-7-7) (a) Active disease burden (*A* + *D*_*i*_), (b) health care system burden ($D_{g}+T_{r}$), (c) non-adherent population ($T_{d}+N_{c}$). See text for more details.

Fig 7 demonstrates the pattern in the disease burden when we consider no treatment delay (i.e., $v^{\prime }=0$). We considered a similar parameter regime (β=11/365, and patient delay nearly 57 days from the onset of symptoms), except that individuals go for treatment immediately after proper diagnosis. There is a major difference in the burden of health care systems and non-adherent populations compare to earlier scenarios (Fig 6). For instance, in the previous case, the earlier burden for non-adherence is $5.01276\times 10^{5}$, now, in the current scenario, it is decreased by 42% (Fig 7(c)). Taken all together, this analysis and simulations underscore that delaying proper diagnosis and treatment of TB disease through multiple consultations without going to DOTS centres can have an enormous impact on society by increasing the disease burden of TB infection as well as the cost of the health care system.

**Fig 8:**
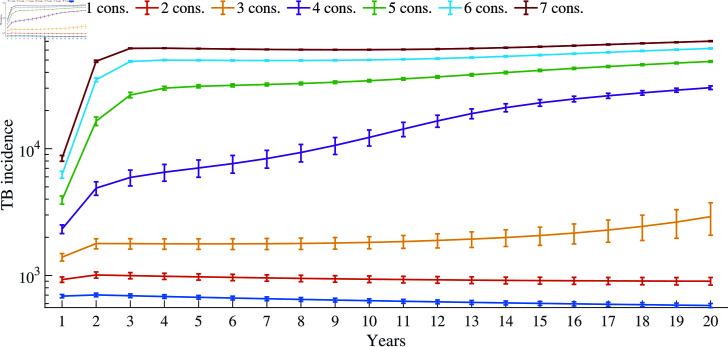
Estimated incidence of TB over 20 years when the number of days between subsequent consultations is 15-7-7-7-7-7-7.

Fig 8 illustrates the estimated increase in new TB cases over a 20-year period, attributed to individuals seeking multiple consultations and switching providers before attending a DOTS center. As shown, the TB burden starts to rise gradually a few years after the third consultation and remains consistently high if each exposed person visits the DOTS centre after five or more consultations. This pattern may explain the recent non-decreasing trend in TB incidence observed in India and potentially in other countries (see Fig 1).

## Sensitivity analysis

**Fig 9:**
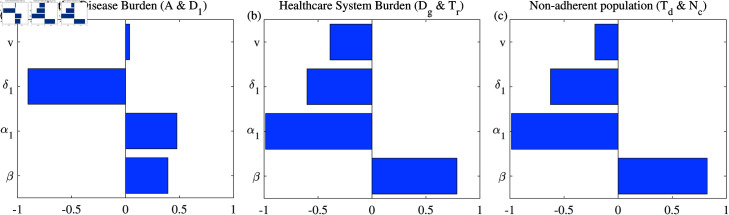
Tornado plot (obtained using PRCC) exhibits influence of crucial parameters on the dynamics of Active disease burden (a), Healthcare system burden (b) and Non-adherent population (c). The baseline parameter values are given in Table 2 with $\alpha_{1}=0.5$, $\alpha_{2}=1\;-\;\alpha_{1}$, $\delta_{1}=1/7$, $\delta_{2}=1/30$, $\eta_{1}=0.1$, $\eta_{2}=0.5$, $\eta_{3}=0.03$. We conducted 1000 simulations with the parameter ranges: β from 20/365 to 30/365, $\alpha_{1}$ from 0 to 1, $\delta_{1}$ from 1/14 to 1/5 and *v* from 0.70 to 0.85. We have used ODE45 to simulate the model for 700 days and the end point is taken to perform the sensitivity analysis.

We computed partial rank correlation coefficients (PRCC) and performed a sensitivity analysis to evaluate parameter uncertainties. We consider parameters such as average number of infections per TB case per day (β), treatment-seeking rate in first consultations $\alpha_{1}$, duration until proper diagnosis at DOTS centre ($1/\delta_{1}$) and immediate treatment initiation rate (*v*) on model outcomes [54]. For simplicity, we reduced the number of consultations from seven to two. PRCC quantifies the strength of the relationship between the model outcomes and those parameters, indicating the degree of influence each parameter has on the outcomes. The results of the sensitivity analysis for active disease burden (Fig 9(a)) suggest that it is affected to a moderate degree by β, as evidenced by the PRCC of  + 0.4. This suggests that as transmission rates increase, the number of active tuberculosis cases also increases. Although $\alpha_{1}$ exhibits a positive correlation (PRCC of +0.5) with total burden in *A* & *D*_1_ compartments (due to increase in proportion population in *D*_1_ with higher rate of $\alpha_{1}$), it has negative impact (PRCC (= -1)) on healthcare system and non-adherent population. There exists a robust inverse relationship between $\delta_{1}$ and the active disease burden (PRCC = –0.9 and –0.5), and other compartments as well, suggesting that earlier diagnosis significantly mitigates the burden of the disease. The minor positive influence of *v* (PRCC = +0.05) on active cases is attributable to its comparatively lower immediate impact in comparison to other parameters. Taken together, the sensitivity analysis highlights the critical importance of controlling the transmission rate, ensuring early diagnosis, and reducing the duration until diagnosis to effectively manage TB. Encouraging treatment-seeking behaviour after diagnosis further supports these efforts by maintaining adherence and reducing strain on healthcare systems. These insights point to key areas for intervention in TB control strategies.

## Optimal control model

### Model and objective function formulation

Our simulations and analyses highlight the significant impact of patient delay, diagnosis delay, and treatment delay on TB incidence within the population. Building on this understanding, we propose an optimal control problem incorporating targeted interventions [55]. In optimal control analysis, we assume number of consultations is two, instead of seven. In our optimal control framework, we introduced two control variables: *u*_1_ - represents targeted campaigns aimed at increasing awareness of TB symptoms, promoting early detection, and educating communities about TB transmission and care. *u*_2_ - focuses on addressing healthcare system challenges through *public-private engagement*, such as implementing strategic measures to improve medical infrastructure, ensuring timely and efficient diagnosis, and facilitating effective TB treatment. The optimal control model, incorporating these strategic interventions and their associated control variables, is described by the following system of differential equations:


$$\frac{dS}{dt}=\Lambda -\lambda S-\mu S$$

$$\frac{dA}{dt}=\lambda S-(\alpha_{1}+\alpha_{2}u_{1}+\alpha_{2}(1-u_{1}))A+rR-(\mu +\mu_{d})A$$

$$\frac{dD_{1}}{dt}=(\alpha_{1}+\alpha_{2}u_{1})A-\delta_{1}(1+u_{2})D_{1}$$

$$\frac{dD_{2}}{dt}=\alpha_{2}(1-u_{1})A-\delta_{2}(1+u_{2})D_{2}$$

$$\frac{dD_{g}}{dt}=\delta_{1}(1+u_{2})D_{1}+\delta_{2}(1+u_{2})D_{2}-(v+v\prime )D_{g}-(\mu +\mu_{d})D_{g}$$

$$\frac{dT_{r}}{dt}=vD_{g}+wT_{d}-(w\prime +\gamma )T_{r}-(\mu +\mu_{d})T_{r}$$

$$\frac{dT_{d}}{dt}=v\prime D_{g}+nN_{c}-wT_{d}-(\mu +\mu_{d})T_{d}$$

$$\frac{dN_{c}}{dt}=w\prime T_{r}-nN_{c}-(\mu +\mu_{d})N_{c}$$

$$\frac{dR}{dt}=\gamma T_{r}-(r+\mu )R$$

where the force of infection (λ) is given by


$$\lambda =\frac{\beta S(A+D_{1}+D_{2}+\eta_{1}D_{g}+\eta_{2}T_{d}+\eta_{3}T_{r}+\eta_{4}N_{c})}{N}$$

Here, $\eta_{i}$ represents the reduced probability of transmission of infection for diagnosed, diagnosed but delaying treatment, treated, and non-compliant individuals.

Our main goal in this study is to minimize total cost *J* defined below under optimal interventions *u*_1_ and *u*_2_. We assume ξ defines the per capita treatment cost, which reflects costs required to diagnosis and treat patients at the DOTS centres. *c*_1_ which represents the per capita cost of organizing campaigns aimed at educating the public about TB disease, its symptoms, and the importance of early case detection. It encompasses expenses associated with organizing community events, distributing informational materials, and utilizing media platforms to effectively disseminate TB-related information, and the cost *c*_2_ which reflects per capita cost due to improving medical facilities to mitigate delays in the healthcare system. This encompasses expenditures associated with the enhancement of diagnostic infrastructure, the expansion of medical apparatus availability, etc. Based on this, we have considered a quadratic objective function:

$$J(u_{1},u_{2})=\min\int_{0}^{T}[\xi (A+D_{1}+D_{2})+\frac{c_{1}u_{1}^{2}}{2}+\frac{c_{2}u_{2}^{2}}{2}]\;dt$$ (2)

where $A,D_{1},D_{2}$ are the model variables defined above. Thus, we pursue optimal control analysis to find out $u_{1}^{*}$ and $u_{2}^{*}$ for our objective function such that


$$J(u_{1}^{*},u_{2}^{*})=\min\{J(u_{1},u_{2}):u_{1}\in \Omega_{1},u_{2}\in \Omega_{2}\}\;$$

where $\Omega_{1}$ and $\Omega_{2}$ are defined by


$$\Omega_{1}=\{u_{1}:0\leq u_{1}(t)\leq 1,t\in [0,T]\}$$

$$\Omega_{2}=\{u_{2}:0\leq u_{2}(t)\leq \bar{u_{2}},t\in [0,T]\}$$

where

$$u_{2}<\bar{u_{2}}:=\min(\frac{1}{\delta_{1}}-1,\frac{1}{\delta_{2}}-1)$$ (3)

where $1/\delta_{i}$ represents the average period individuals spent in *D*_*i*_ compartments before actually going to the DOTS centre for diagnosis.

### Existence of optimal solution

**Theorem 1.** *There exist optimal controls* $u_{1}^{*}\in \Omega_{1}$ *and* $u_{2}^{*}\in \Omega_{2}$ *and solutions* $S^{*},A^{*},D_{1}^{*}$, $D_{2}^{*},D_{g}^{*},T_{r}^{*},T_{d}^{*},N_{c}^{*},R^{*}$ *such that* $J(u_{1}^{*},u_{2}^{*})$= $\min J(u_{1},u_{2})$ *over* [0,*T*]. *Further, there exist piecewise differentiable adjoint variables* $\psi_{i}$ *where* i=1 to 9*, satisfying*


$$\frac{d\psi_{i}}{dt}=-\frac{\partial H(t,S^{*},A^{*},D_{1}^{*},D_{2}^{*},D_{g}^{*},T_{r}^{*},T_{d}^{*},N_{c}^{*},R^{*},u_{1}^{*},u_{2}^{*},\psi_{i})}{\partial i}$$

*where* i=1…9 *and transversality condition*


$$\psi_{1}^{*}(T)=\psi_{2}^{*}(T)=\psi_{3}^{*}(T)=\psi_{4}^{*}(T)=\psi_{5}^{*}(T)=\psi_{6}^{*}(T)$$

$$=\psi_{7}^{*}(T)=\psi_{8}^{*}(T)=\psi_{9}^{*}(T)=0$$

*Proof*: The integrand $\xi (A+D_{1}+D_{2})+\frac{c_{1}u_{1}^{2}}{2}+\frac{c_{2}u_{2}^{2}}{2}$ in eq (2) is convex with respect to *u*_1_(*t*) and *u*_2_(*t*) on the control sets $\Omega_{1}$ and $\Omega_{2}$, respectively. Both the control variables $u_{1}(t)\in \Omega_{1}$ and $u_{2}(t)\in \Omega_{2}$ are closed and bounded (by the definition). Also, it satisfies the Lipchitz condition with respect to the state variables. Thus, the optimal controls exist that minimizes the objective function defined in Eq 2. We apply the Pontryagin Maximum Principle (PMP) in order to obtain the necessary conditions for optimal control functions. The Hamiltonian


$$H(t,(S,A,D_{1},D_{2},D_{g},T_{r},T_{d},N_{c},R)(t),\psi_{i}(t))$$

is constructed from model equations and objective function as follows:

$$H=\xi (A+D_{1}+D_{2})+\frac{c_{1}u_{1}^{2}}{2}+\frac{c_{2}u_{2}^{2}}{2}\;+\psi_{1}(\Lambda -\lambda S-\mu S)\;+\psi_{2}(\lambda S-(\alpha_{1}+\alpha_{2}u_{1}+\alpha_{2}(1-u_{1}))A+rR-(\mu +\mu_{d})A)\;+\psi_{3}((\alpha_{1}+\alpha_{2}u_{1})A-\delta_{1}(1+u_{2})D_{1})\;+\psi_{4}(\alpha_{2}(1-u_{1})A-\delta_{2}(1+u_{2})D_{2})\;+\psi_{5}(\delta_{1}(1+u_{2})D_{1}+\delta_{2}(1+u_{2})D_{2}-(v+v\prime )D_{g}-(\mu +\mu_{d})D_{g})\;+\psi_{6}(vD_{g}+wT_{d}-(w\prime +\gamma )T_{r}-(\mu +\mu_{d})T_{r})\;+\psi_{7}(v\prime D_{g}+nN_{c}-wT_{d}-(\mu +\mu_{d})T_{d})\;+\psi_{8}(w\prime T_{r}-nN_{c}-(\mu +\mu_{d})N_{c})\;+\psi_{9}(\gamma T_{r}-(r+\mu )R)$$ (4)

where $\psi_{i},\;\;i=1,2,\ldots ,9$ are associated adjoint variables for the states $S,A,D_{1},D_{2},D_{g},T_{r},T_{d},N_{c},R$ respectively given by the canonical equations


$$\frac{d\psi_{1}}{dt}=-\frac{\partial H}{\partial S},\frac{d\psi_{2}}{dt}=-\frac{\partial H}{\partial A},\frac{d\psi_{3}}{dt}=-\frac{\partial H}{\partial D_{1}},$$

$$\frac{d\psi_{4}}{dt}=-\frac{\partial H}{\partial D_{2}},\frac{d\psi_{5}}{dt}=-\frac{\partial H}{\partial D_{g}},\frac{d\psi_{6}}{dt}=-\frac{\partial H}{\partial T_{r}},$$

$$\frac{d\psi_{7}}{dt}=-\frac{\partial H}{\partial T_{d}},\frac{d\psi_{8}}{dt}=-\frac{\partial H}{\partial N_{c}},\frac{d\psi_{9}}{dt}=-\frac{\partial H}{\partial R}$$ (5)

with transversality conditions $\psi_{i}^{*}=0$.

Substituting the Hamiltonian value gives the canonical system as follows:


$$\frac{d\psi_{1}}{dt}=\frac{\beta }{N}(\psi_{1}-\psi_{2})(A+D_{1}+D_{2}+\eta_{1}D_{g}+\eta_{2}T_{d}+\eta_{3}T_{r}+\eta_{4}N_{c})+\psi_{1}\mu$$

$$\frac{d\psi_{2}}{dt}=-\xi +\frac{\beta S}{N}(\psi_{1}-\psi_{2})+(\psi_{2}-\psi_{3})(\alpha_{1}+\alpha_{2}u_{1})+(\psi_{2}-\psi_{4})(\alpha_{2}(1-u_{1}))+\alpha_{2}(\mu +\mu_{d})$$

$$\frac{d\psi_{3}}{dt}=-\xi +\frac{\beta S}{N}(\psi_{1}-\psi_{2})+(\psi_{3}-\psi_{5})(\delta_{1}(1+u_{2}))$$

$$\frac{d\psi_{4}}{dt}=-\xi +\frac{\beta S}{N}(\psi_{1}-\psi_{2})+(\psi_{4}-\psi_{5})(\delta_{2}(1+u_{2}))$$

$$\frac{d\psi_{5}}{dt}=\frac{\beta S}{N}(\psi_{1}-\psi_{2})\eta_{1}+(\psi_{5}-\psi_{6})v+(\psi_{5}-\psi_{7})v\prime +\psi_{5}(\mu +\mu_{d})$$

$$\frac{d\psi_{6}}{dt}=\frac{\beta S}{N}(\psi_{1}-\psi_{2})\eta_{3}+(\psi_{6}-\psi_{8})w\prime +(\psi_{6}-\psi_{9})\gamma +\psi_{6}(\mu +\mu_{d})$$

$$\frac{d\psi_{7}}{dt}=\frac{\beta S}{N}(\psi_{1}-\psi_{2})\eta_{2}+(\psi_{7}-\psi_{6})w+\psi_{7}(\mu +\mu_{d})$$

$$\frac{d\psi_{8}}{dt}=\frac{\beta S}{N}(\psi_{1}-\psi_{2})\eta_{4}+(\psi_{8}-\psi_{7})n+\psi_{8}(\mu +\mu_{d})$$

$$\frac{d\psi_{9}}{dt}=(\psi_{9}-\psi_{2})r+\psi_{9}\mu$$

Now, to obtain the optimal controls $u_{1}^{*}$ and $u_{2}^{*}$ which minimizes *J* over the regions $\Omega_{1}$ and $\Omega_{2}$ , we use the Hamiltonian minimization condition $\frac{\partial H}{\partial u_{1}}=0=\frac{\partial H}{\partial u_{2}}$, for *u*_1_ and *u*_2_, we have-


$$u_{1}^{*}=\frac{A(\psi_{4}-\psi_{3})\alpha_{2}}{c_{1}}$$

$$u_{2}^{*}=\frac{(\psi_{3}-\psi_{5})\delta_{1}D_{1}+(\psi_{4}-\psi_{5})\delta_{2}D_{2}}{c_{2}},$$

which depend on solutions of adjacent system in $\psi_{i}$. ◻

We now simulate the optimal control model using forward-backward sweep method [55] under different scenarios of parameter regime.

#### Optimal solutions under higher transmission potentiality:

**Fig 10:**
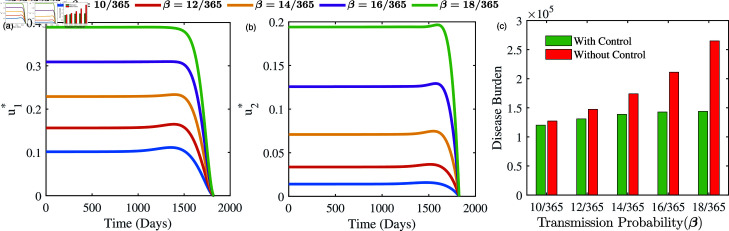
Optimal solution of intervention efforts ($u_{1}^{*}$ and $u_{2}^{*}$) for different values of transmission potentiality β, which is average number of infections per TB case per day. Figure in the rightmost panel exhibits the disparities in the burden of active TB patients with and without control. See text for more discussion.

Fig 10 exhibits the optimal solutions $u_{1}^{*}$ and $u_{2}^{*}$ under different values of transmission potentiality β, which is average number of infections per TB case per day. As we have seen, the intervention effort (*u*_1_) for campaigning and spreading awareness in the community should be increased as transmission potentiality increases.

Similarly, improving the healthcare infrastructure and public-private engagement (*u*_2_) should also increase with higher β. For example, *u*_1_ and *u*_2_ are rising from 0.1 to 0.4 (Fig 10(a)) and 0.02 to 0.2 (Fig 10(b)) respectively, when β is increased from 0.027 to 0.24. The increase in *u*_1_ highlights the importance of community awareness and early detection programmes. On the other hand, the rise in *u*_2_ indicates the need for strategic interventions to improve medical infrastructure, ensuring prompt and effective diagnosis and treatment of TB in the presence of a higher transmission of TB in the community. The substantial variation in extent of *u*_1_ and *u*_2_ implies that greater importance should be given to the efficacy of targeted campaigns (*u*_1_) in comparison to endeavours intended for enhancing healthcare infrastructures (*u*_2_). Policymakers and public health officials can use this information to prioritize interventions so that targeted campaigns receive a larger investment when allocating resources because these campaigns not only help with early detection but also have a significant impact on community behaviour, which slows the spread of TB. Fig 10(c) emphasizes that when the transmission rate of TB escalates, the overall burden of the disease is substantially diminished when both control measures are enforced. Therefore, the implementation of controls *u*_1_ and *u*_2_ significantly decreases the overall impact of diseases.

#### Optimal solutions under different choices between consultations:

**Fig 11:**
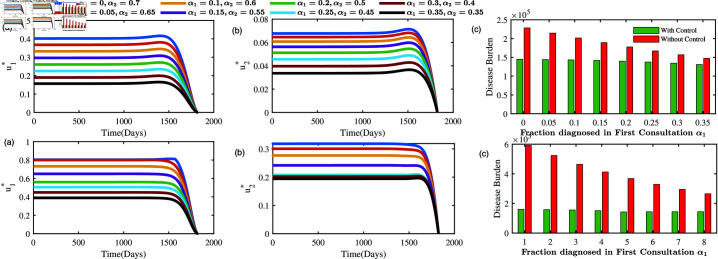
Upper panel: Optimal solutions of intervention efforts $u_{1}^{*}$ (left panel), $u_{2}^{*}$ (right panel) and disease burden (right panel) under different choices of consultation pathways at β=12/365. Lower panel: same for higher transmission probabilities β=18/365.

We also identify and analyse optimal solutions based on varying treatment-seeking behaviours, where individuals choose between one consultation (*D*_1_) and two consultations (*D*_2_) at different rates. Specifically, we consider eight different combinations of ($\alpha_{1},\alpha_{2}$), such as (0,0.7),(0.05,0.65),…,(0.35,0.35), representing different rates at which individuals opt for either one (*D*_1_) or two (*D*_2_) consultations. Fig 11 plots the optimal solutions and disease burdens in respective combinations. Higher rate of multiple consultations (i.e., more than one consultation) increases the amount of controls high. For example, when $\left(\alpha_{1},\alpha_{2})=(0,0.7\right)$, i.e., all active individuals get diagnosed exactly after the second consultation, the optimal *u*_1_ = 0.4. As $\alpha_{1}$ increases and $\alpha_{2}$ decreases such as ($\alpha_{1},\alpha_{2})=(0.35,0.35)$, the control variable *u*_1_ also decreases to 0.15 (Fig 11(upper left panel)). This similar pattern is also observed for the intervention effort *u*_2_ (Fig 11(upper middle panel)). The disease burdens with and without control also decrease as proportion of individuals are diagnosed only after first consultation increases (Fig 11(upper right panel)). Lower panel describes the same result with higher β (=18/365). Both simulations show that we should prioritize targeted campaigns (*u*_1_) over efforts to improve healthcare infrastructure (*u*_2_) by a factor of 10. This analysis offers comparative insights into optimal control strategies for various individual preferences, providing valuable guidance for public health policymakers.

#### Cost sensitivity analysis of optimal controls:

**Fig 12:**
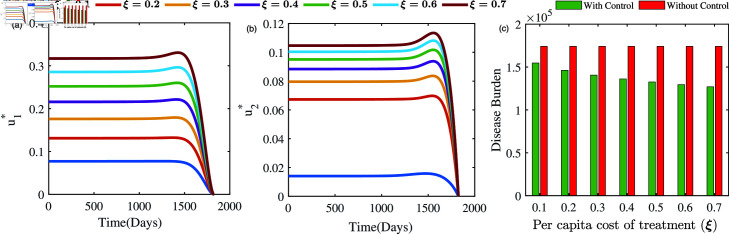
Optimal solutions $u_{1}^{*}$ (left panel) and $u_{2}^{*}$ (right panel) and disease burden (right panel) under different per capita cost of treatment.

The impact of variation in the per capita cost of treatment on the evolution of control strategies *u*_1_ and *u*_2_ is illustrated in Fig 12. It is noteworthy that *u*_1_ retains a moderate value of 0.08 (Fig 12a) as a result of the treatment’s low cost, while *u*_2_ hovers around 0.018 (Fig 12b). In contrast, as treatment costs escalate, *u*_1_ significantly increases to 0.32, surpassing its initial value, while *u*_2_ increases to 0.11. Based on our analysis of these results, we conclude that the significance of *u*_1_ and *u*_2_ is restricted in situations where per capita treatment costs are comparatively low and accessible. As treatment costs rise, there is an urgent need for more effective control measures. The same is also reflected in Fig 12c. This is because higher treatment expenses significantly increase the total cost when dealing with a large number of cases, necessitating stronger control efforts to mitigate the burden. It is also noteworthy to observe *u*_1_ exceeds that of *u*_2_, underscoring the importance of *u*_1_ over the control *u*_2_ in situations involving elevated treatment expenses. Our findings underscore the critical need to prioritize targeted campaigns for effective tuberculosis management, especially in resource-limited settings such as low-income countries, where the high costs of treatment pose a major restriction. This highlights the intricate interplay between control strategies and the considerable economic burden associated with tuberculosis treatment.

**Fig 13:**
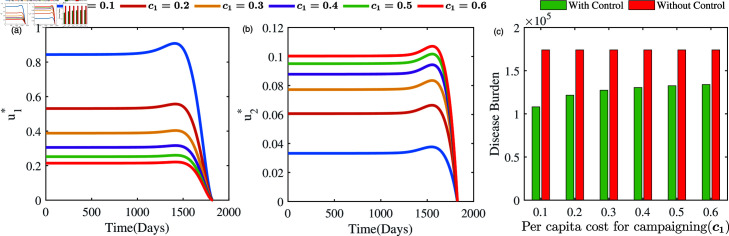
Optimal solutions $u_{1}^{*}$ (left panel) and $u_{2}^{*}$ (right panel) and disease burden (right panel) under different per capita cost of campaigning.

**Fig 14:**
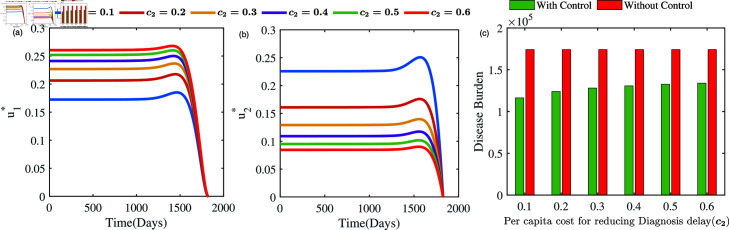
Optimal solutions $u_{1}^{*}$ (left panel) and $u_{2}^{*}$ (right panel) and disease burden (right panel) under different per capita cost for reducing health care system issues.

In our investigation, we also analyse the evolution of *u*_1_ and *u*_2_ under varying per capita campaigning costs (*c*_1_) in efforts to control TB within the community. Fig 13 illustrates the results when *c*_1_ increases while *c*_2_ remains fixed, whereas Fig 14 presents the outcomes when *c*_2_ increases while *c*_1_ remains fixed. Both simulations demonstrate that higher per capita control costs lead to reduced control efforts compared to the other parameter. Specifically, an increase in *c*_1_ results in a lower *u*_1_ and a higher *u*_2_ as optimal, while an increase in *c*_2_ yields a lower *u*_2_ and a higher *u*_1_ as optimal, minimizing the total cost. These findings provide valuable insights for policymakers in designing effective TB control strategies, particularly in low-income and lower-economic settings.

## Discussion and conclusion

Tuberculosis remains one of the most pressing public health crises in South-East Asia and Africa. An immediate diagnosis and treatment is essential to curbing the transmission of this highly contagious disease [56, 57]. However, numerous studies have highlighted that patients often fail to recognize the severity of their symptoms or limited healthcare facilities, leading to delays in seeking medical care [58–60]. In fact, individuals with subclinical or asymptomatic TB may not experience any symptoms, making them unaware of their infection, and they can therefore act as a source of transmission without even knowing they are infectious. Studies suggest that a significant portion of TB transmission may be from asymptomatic TB, contributing to the global TB burden. [61, 62]. Such delays worsen poverty, amplify social inequalities, and drive migration, ultimately reducing productivity and fuelling unstable economic cycles—conditions that further heighten the risk of TB transmission. This perpetuates a continuous cycle of infection and socioeconomic harm, highlighting the urgent need for collective efforts to enhance TB care-seeking, diagnosis, and treatment, thereby alleviating the global burden of the disease.

In our study, we have incorporated the information from the survey studies and developed a mathematical framework to illustrate how multiple consultations by patients prior to the accurate diagnosis of TB affect the propagation of the disease within a population. The conclusions of our stochastic simulation across various parameters indicate substantial consequences in terms of disease burden and burden to the healthcare system. Although we lack direct empirical data for immediate validation, simulations and sensitivity analysis using PRCC demonstrate that the model behaves consistently and realistically under various parametric conditions. For instance, having up to three consultations before visiting a DOTS center may have minimal impact, whereas more than three consultations can significantly increase the disease burden within the community (Fig 8). Additionally, sensitivity analysis reveals that $\alpha_{1}$ and $\delta_{1}$ have strong positive and negative impacts on disease burden, respectively, indicating that a higher rate and longer delays in seeking proper treatment lead to a greater TB burden on the community.

In addition, we also presented an optimal control model designed to find the most efficient interventions, such as targeted campaigns to increase awareness of TB symptoms, facilitate early detection, and adopt strategic actions to improve healthcare infrastructure. Optimal control model findings emphasize that the role of targeted campaigns becomes very critical in response to the severity of the disease’s transmission rate. Timely detection during initial consultations with healthcare practitioners greatly minimizes the overall burden of the disease, underscoring the need of early diagnosis in minimizing its adverse effects.

While results from our research provides valuable insights of delayed diagnosis in TB disease, there are also limitations of our model. We have considered the sojourn times in ordinary differential equations (ODEs) as patient delay due to multiple consultations. However, sojourn times in ODEs are often implicitly assumed to follow an exponential distribution, which means that the system has a Markovian property (memoryless transitions). However, this behavioural interaction due to multiple consultations can also be modelled by explicitly incorporating delays, which is non-exponential. It can be modelled by incorporating fixed or distributed delays, framing as Delay Differential Equations (DDEs) or Integro-Differential Equations (IDE), which are also non-Markovian. There are various modelling studies using DDEs and IDEs in infectious disease epidemiology [63, 64]. One can also explore an age-structured model that incorporates disease-induced mortality of elderly patients during multiple consultations under different HCPs. The impact of stigma on treatment-seeking behaviour for tuberculosis is also one significant factor that needs more investigation. Developing modelling framework including fear of discrimination, social isolation, and misconceptions may be one step further improvement of the current research in modelling TB. Our model also assumes homogeneity in patient behaviour and healthcare access. Also, it does not account for individual-level interactions and heterogeneity in disease transmission dynamics. So, models including age-structure or social structure may be a good opportunity in modelling treatment seeking behaviour in TB and its impact.

Nonetheless, our model could establish a platform for discussing the treatment-seeking behaviour of tuberculosis (TB) patients and its impact on the healthcare system’s burden. These insights could furnish policymakers and public health authorities with a blueprint for effectively allocating resources to combat TB, ensuring that targeted initiatives receive adequate funding. Awareness campaigns and public health strategies should aim to improve public awareness about the possibility of asymptomatic TB and the importance of seeking medical attention even in the absence of symptoms. Additionally, optimal control analysis demonstrates that implementing proper control measures leads to significant reductions in the overall disease burden, especially in regions experiencing escalating TB transmission rates. Despite potential obstacles like rising treatment expenses, adopting these targeted interventions may lead to noticeable enhancements in community-level disease management effort.
